# Application of flap economics in head and neck reconstruction

**DOI:** 10.1016/j.jpra.2025.08.022

**Published:** 2025-08-28

**Authors:** Wen Peng, Haixia Zhang, Lei Jin, Zan Li, Chuanzheng Sun

**Affiliations:** a Department of Head and Neck Surgery, The Third Affiliated Hospital of Kunming Medical University, Yunnan Cancer Hospital, Kunming, Yunnan, China; b Department of Oncology, Plastic Surgery, Hunan Cancer Hospital, The Affiliated Cancer Hospital of Xiangya School of Medicine, Central South University, Changsha, Hunan, China

**Keywords:** Kiss flap, Head and neck reconstruction, Free flap, Pedicle flap, Autologous transplantation

## Abstract

**Background:**

The Kiss flap technique, aligned with flap economics, has gained traction in head and neck cancer reconstruction. This study evaluates “classic” and “non-classic” Kiss flap methods and explores flap economics principles.

**Methods:**

We retrospectively analyzed 50 patients undergoing head and neck cancer surgery with Kiss flap reconstruction between January 2016 and May 2023.

**Results:**

Patients (34 males, 16 females; mean age 53) were categorized into “classic” (*n* = 27) and “non-classic” (*n* = 23) groups. Seventy percent exhibited good postoperative healing. The anterolateral thigh (ALT) flaps showed the highest stability and lowest complication rate (21 %), followed by radial forearm free flap (RFFF) (30 %), pectoralis major myocutaneous flap (PMMF) (46 %), and Fibula (50 %).

**Conclusion:**

Both Kiss flap techniques represent viable alternative options for head and neck reconstruction in complicated cases. ALT flaps offer superior stability. Flap economics aids in resource optimization, emphasizing personalized flap selection for optimal outcomes.

## Introduction

Postoperative reconstruction of head and neck cancer remains a significant challenge for modern surgeons. The complex anatomy and diverse functions of this region often result in intricate defects following radical tumor resection, affecting patients’ appearance and vital functions, thereby impacting their quality of life.^1^, ^2^, ^3^

The principle of Economic Autologous Tissue Transfer aims to maximize the use of a patient’s own tissue while minimizing donor site morbidity. In 2016, Zhang et al.^4^ introduced the “kiss flap technique,” classifying flaps based on their blood supply into single, multiple, and random types. The “classic” Kiss flap technique involves converting a single flap with multiple blood supplies into smaller skin paddles, which are then arranged in a “kissing” pattern to optimize the coverage of complex defects. However, the “non-classic” Kiss flap technique, which involves de-epithelialization of single perforator or musculocutaneous flaps, was based on our clinical observations and applications. The “non-classic” Kiss flap achieves donor-site economy by optimizing harvest dimensions for direct closure, streamlining perforator dissection to enhance operative efficiency, and improving long-term aesthetic outcomes in cosmetically sensitive regions. “Non-classic” Kiss flaps are indicated for critical scenarios: salvage reconstruction of through-and-through defects, where dual-surface repair is achieved via targeted de-epithelialization, and defect repair using thinned forearm flaps, where geometric reconfiguration (length-to-width conversion) facilitates primary donor-site closure while maintaining perfusion reliability. This approach synergizes functional restoration and aesthetic optimization, particularly in cases demanding thin, pliable tissue.

While both “classic and non-classic” Kiss flaps achieve primary donor-site closure through geometric reconfiguration, “classic” Kiss flaps prioritize enhanced perfusion reliability and intraoperative flexibility for dual-layer repairs. In contrast to conventional flaps, “non-classic” variants excel in reconstructing complex defects by enabling personalized reconstruction protocols via single-perforator-based modular designs, while eliminating the need for secondary donor-site grafting.

The anterolateral thigh (ALT) flap is a widely used workhorse flap in head and neck reconstruction.^5^, ^6^, ^7^, ^8^, ^9^ The fibula flap (Fibula), radial forearm free flap (RFFF), and pectoralis major myocutaneous flap (PMMF) also serve as common options.^10^, ^11^, ^12^, ^13^, ^14^

Comparative studies on kiss flap applications in head and neck reconstruction remain scarce, especially those providing comprehensive multi-center, site, and flap type analyses. This study aims to: evaluate the efficacy and safety of both “classic and non-classic” Kiss flap techniques in head and neck reconstruction; compare outcomes across four different flap types (ALT, RFFF, PMMF, and Fibula); and provide evidence-based guidance for individualized reconstruction strategies.

## Materials and methods

### Materials

This study retrospectively analyzed clinical data from 50 patients who underwent radical head and neck cancer surgery at Yunnan Cancer Hospital and Hunan Cancer Hospital between January 2016 and May 2023.

Postoperative defects were categorized into three types. Type A (Skin defects): Scalp and cervicofacial skin defects. Type B (Oral and maxillofacial defects): Localized oral cavity defects or combined defects involving the oral cavity with jawbone or midface (nasal cavity, paranasal sinuses, and orbit). Type C (Hypopharyngeal and below defects): Defects of the hypopharynx, larynx, or cervical esophagus.

Inclusion criteria: ① Defect types A, B, or C; ② Radical head and neck cancer resection with reconstruction using ALT, RFFF, PMMF, or Fibula; ③ Application of Kiss flap technique.

Exclusion criteria: ① Stage I patients (T1N0M0); ② Recurrence after radical resection; ③ Preoperative radiotherapy history; ④ Incomplete case data.

### Surgical procedure

Both centers adhered to identical surgical protocols, defined as standardized workflows integrating radical tumor resection with flap economy principles to achieve simultaneous oncologic control and reconstructive outcomes. Two surgical teams collaborated: one for primary tumor resection and intraoperative frozen pathological examination, ensuring negative margins, and determining the extent of neck lymph node dissection based on tumor location and clinical findings. The other team prepared flaps and performed reconstruction.

The Kiss flap technique application varied by flap type and blood supply. For flaps with multiple blood supply sources, the “classic” method converted the length into the required width or shape, assembling multiple skin islands. For single perforator or musculocutaneous flaps, a “non-classic” approach designed the flap shape according to the defect, followed by careful de-epithelialization to avoid compromising blood supply. The de-epithelialized flap was then manipulated as needed to create the Kiss flap. Flap crisis is defined as an emergent clinical condition characterized by arterial insufficiency, impaired venous drainage, or systemic threats to flap viability during or after transplantation, mandating immediate intervention to prevent partial or total necrosis.

### Statistical analysis

Continuous data were presented as mean ± standard deviation (M ± SD), and categorical data as relative composition ratios ( %). Statistical analyses were conducted using SPSS version 26.0. Depending on normality and variance homogeneity, continuous data were analyzed with independent sample *t*-tests, Kruskal-Wallis H tests, or Mann-Whitney U tests. Categorical data comparisons employed the χ² test or Fisher’s exact test, with a *P*-value of <0.05 indicating statistical significance.

## Results

In this study, 50 patients participated, including 34 males and 16 females, with an average age of 53.10 years (range 25–79). Patients were stratified into “classic” (*n* = 27) and “non-classic” (*n* = 23) groups based on the Kiss flap harvesting technique. No significant differences were observed in age, sex, or tumor stage between the groups (Table 1), and none had distant metastasis.

**Table 1:** Clinical characteristics of patients.

		Total (*n* = 50)	Classic (*n* = 27)	Non-classic (*n* = 23)	*P*-value
Age(year, M ± SD)		53.10 ± 12.17	55.04 ± 13.40	50.83 ± 10.38	0.226
Gender(%)	male	34(68.0)	19(70.4)	15(65.2)	0.697
	female	16(32.0)	8(29.6)	8(34.8)	
T-stage(%)	T2	19(38.0)	6(22.2)	13(56.5)	0.067
	T3	16(32.0)	12(44.4)	4(17.4)	
	T4	15(30.0)	9(33.3)	6(26.1)	
N-stage(%)	N0	31(62.0)	15(55.6)	16(69.6)	0.315
	N1	13(26.0)	8(29.6)	5(21.7)	
	N2	4(8.0)	3(11.1)	1(4.3)	
	N3	2(4.0)	1(3.7)	1(4.3)	
Defect type(%)	A	6(12.0)	6(22.2)	0(0)	0.000
	B	39(78.0)	16(59.3)	23(100)	
	C	5(10.0)	5(18.5)	0(0)	
Flap type(%)	ALT	29(58.0)	21(77.8)	8(34.8)	0.000
	PMMF	9(18.0)	6(22.2)	3(13.0)	
	RFFF	10(20.0)	0(0)	10(43.5)	
	Fibula	2(4.0)	0(0)	2(8.7)	
Flap size	Total	73.24 ± 36.82	89.32 ± 7.60	54.37 ± 4.61	0.000
(cm^2^, M ± SD)	Bigger	39.44 ± 22.23	48.68 ± 4.79	28.60 ± 2.48	0.001
	Smaller	29.23 ± 14.02	34.60 ± 2.88	22.92 ± 2.04	0.002
Complication(%)	None	35(70.0)	20(74.1)	15(65.2)	0.502
	Flap crisis	9(18.0)	3(11.1)	6(26.1)	
	Delayed healing	4(8.0)	3(11.1)	1(4.3)	
	Others	2(4.0)	1(3.7)	1(4.3)	

A, skin defects; B, oral and maxillofacial defects; C, hypopharyngeal and below defects; ALT, anterolateral thigh; RFFF, radial forearm free flap; PMMF, pectoralis major myocutaneous flap.

The “classic” group utilized multi-perforator flaps like the ALT and PMMF, effective for diverse head and neck defect reconstructions. Conversely, the “non-classic” group, which included all four flap types (ALT, PMMF, RFFF, and Fibula), restricted by flap size and mobility, mainly addressed type B defects. Thirty-five patients (70 %) exhibited uneventful postoperative healing. Complications included flap crisis (*n* = 9, 18 %), delayed healing (*n* = 4, 8 %), and others (*n* = 2, 4 %; seroma and pulmonary infection). No statistically significant difference in complication rates was observed between groups.

Postoperative defects varied in head and neck cancer cases (Table 2). Statistical analysis indicated type A defects required the largest average flap size of 104.33 cm² (range 12.0–20.0 cm × 6.0–8.0 cm), ranking first. Type C defects, often involving combined repairs, had an average flap size of 81.70 cm² (range 11.0–19.0 cm × 4.5–8.0 cm), while type B defects needed the smallest at 67.37 cm² (range 6.0–25.0 cm × 2.5–9.0 cm). The flap size differences between defect types were statistically significant (*P* = 0.024).

**Table 2:** Kiss flap size of different locations.

	Flap size(cm^2^, M ± SD)		
	Donor flap	Bigger kiss flap	Smaller kiss flap
A	104.33 ± 31.30	50.41 ± 25.06	42.92 ± 12.93
B	67.37 ± 35.52	36.35 ± 20.18	27.26 ± 13.55
C	81.70 ± 29.40	50.40 ± 31.22	29.40 ± 13.20
*P*-value	0.024	0.313	0.041

A, skin defects; B, oral and maxillofacial defects; C, hypopharyngeal and below defects.

Table 3 details average flap sizes: ALT at 85.52 cm², PMMF at 77.23 cm², RFFF at 32.80 cm², and Fibula at 90.00 cm². Approximately 75 % of ALT flaps were multi-perforator, with 21 harvested using the “classic” and 8 using the “non-classic” approach (including 2 cases with close dual perforators using de-epithelialization). PMMF were pedicled flaps, some combined with thoracoacromial artery perforator (TAAP) flap; 6 cases used “classic” and 5 “non-classic” methods. All RFFF (*n* = 10) and Fibula (*n* = 2) cases were single-perforator flaps using “non-classic” approaches. Kiss flap harvesting methods differed significantly among flap types (*P* = 0.000).

**Table 3:** Characteristics of different flaps.

		ALT(*n* = 29)	PMMF(*n* = 11)	RFFF(*n* = 10)	Fibula(*n* = 2)	*P*-value
Flap size(cm^2^, M ± SD)		85.52 ± 37.61	77.23 ± 26.53	32.80 ± 4.30	90.00 ± 0.00	0.000
Technique(%)	Classic	21(72.4)	6(54.5)	0(0)	0(0)	0.000
	Non-classic	8(27.6)	5(45.5)	10(100.0)	2(100.0)	
Defect type(%)	A	6(20.7)	0(0)	0(0)	0(0)	0.020
	B	22(75.9)	5(55.6)	10(100)	2(100)	
	C	1(3.4)	4(44.4)	0(0)	0(0)	
Complication	None	23(79.3)	6(54.5)	7(70.0)	1(50.0)	0.247
(%)	Flap crisis	4(13.9)	2(18.2)	2(20.0)	1(50.0)	
	Delayed healing	1(3.4)	3(27.3)	0(0)	0(0)	
	Others	1(3.4)	0(0)	1(10.0)	0(0)	

ALT, anterolateral thigh; RFFF, radial forearm free flap; PMMF, pectoralis major myocutaneous flap.

**Figure 1:**
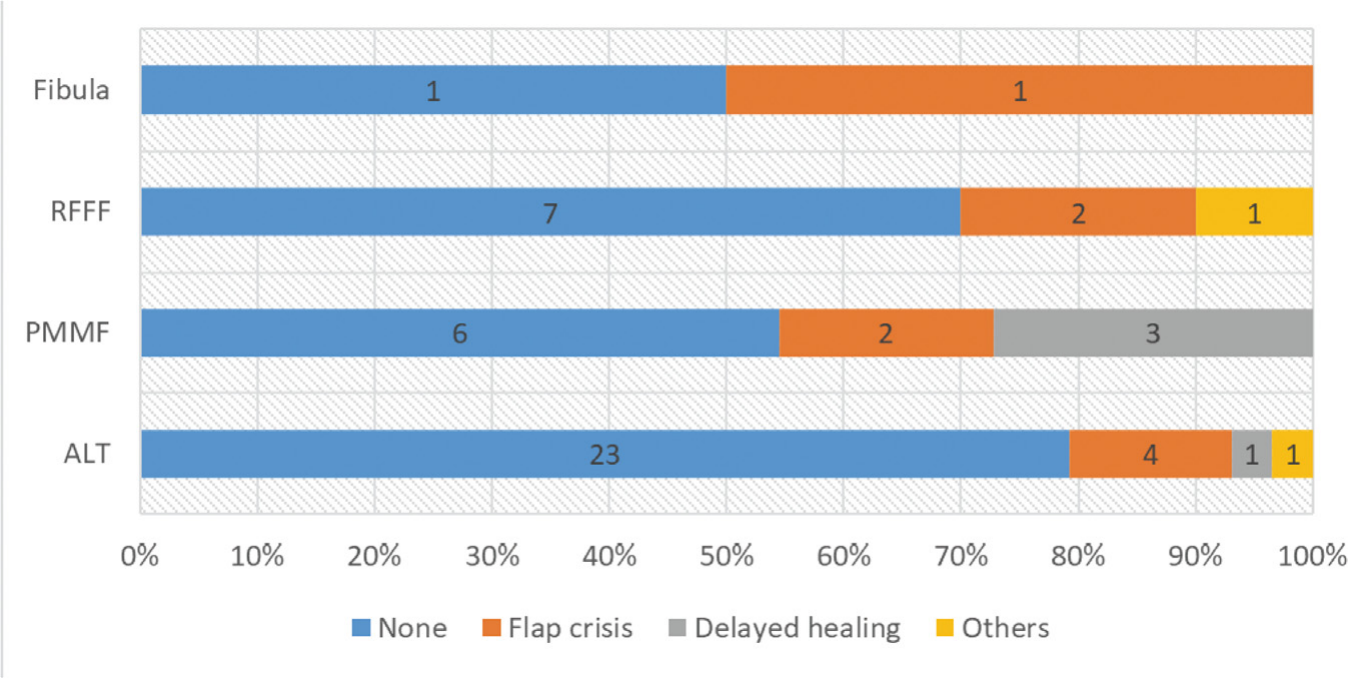
Postoperative complications of different flaps. RFFF: radial forearm free flap. PMMF, pectoralis major myocutaneous flap. ALT, anterolateral thigh.

Among 50 patients, 9 encountered flap crises needing surgical exploration (17 % rate), with 2 failures (1 ALT, 1 Fibula). Both were salvaged by PMMF kiss flaps via the “non-classic” technique, analyzing a total of 52 flaps with a 96 % survival rate: ALT 97 %, Fibula 50 %, RFFF and PMMF 100 %. ALT demonstrated the lowest complication rate (21 %, 6/29), followed by RFFF (30 %, 3/10), PMMF (46 %, 5/11), and Fibula (50 %, 1/2). However, these differences were not statistically significant (*P* = 0.247, Figure 1).

**Figure 2:**
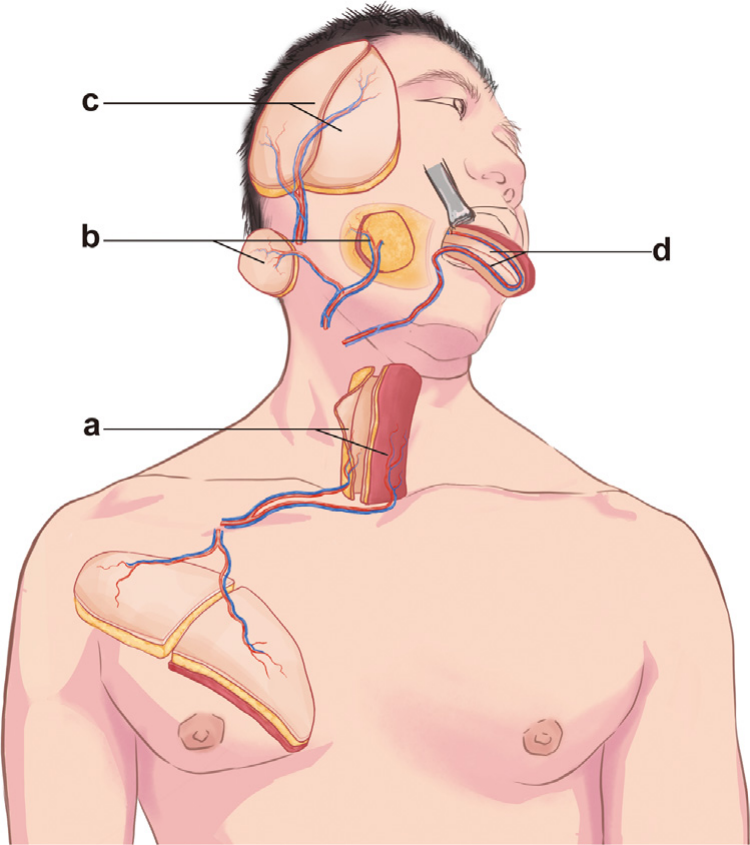
Schematic diagram of KISS flap reconstruction in the head and neck region. (a) “Classic” Kiss flap tubularized PMMF combined with TAAP for the reconstruction of the hypopharyngeal defect. (b) “Classic” ALT kiss flap for the reconstruction of the buccal through-and-through defect. (c) “Classic” ALT Kiss flap for the reconstruction of scalp defect. (d) “Non-Classic” nRFFF kiss flap for the reconstruction of tongue defect.

Finally, we systematically present four representative cases of head and neck reconstruction, highlighting the clinical applications of both “classic” and “non-classic” kiss flap techniques across various anatomical sites (Figure 2). We present the four most commonly used Kiss flaps in complex head and neck reconstruction in a reader-friendly format.

### Clinical reports

#### Case 1

**Figure 3:**
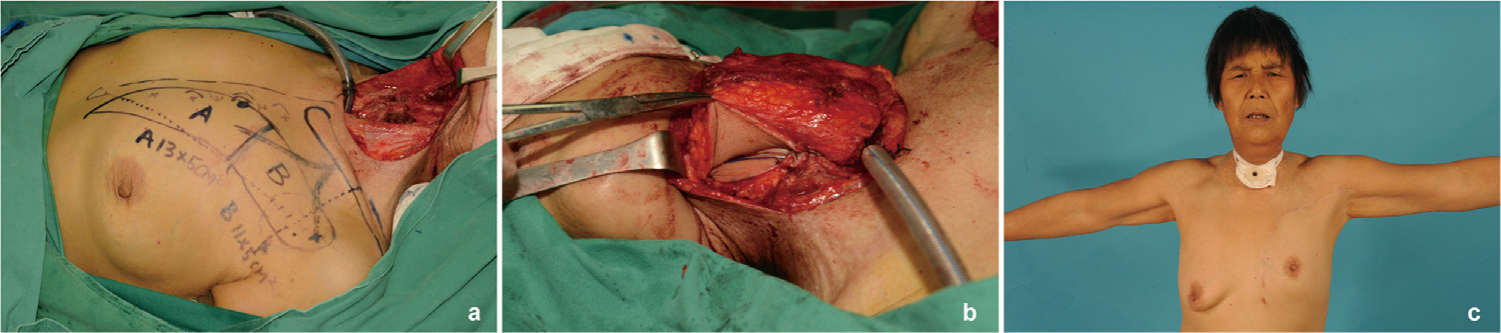
Case 1: Reconstruction of hypopharyngeal segmental defect with pectoralis major myocutaneous flap (PMMF) and thoracoacromial artery perforator (TAAP) flap in the “classic” way. (a) Intraoperative harvesting of the PMMF combined with the TAAP flap in the “classic” way. (b) Reconstruction of the hypopharyngeal defect using the kiss flap. (c) Long-term appearance of neck and donor site at 2.5 years postoperatively.

A 62-year-old female with T3N0M0 moderately differentiated squamous cell carcinoma of the hypopharynx underwent left functional neck dissection, total laryngectomy, and cervical esophagus resection. Post-resection, a typical circumferential defect was present in the neck, approximately 8 cm long, extending from the tongue base to the upper sternal notch. A “classic” kiss flap was prepared using a left thoracoacromial artery perforator (TAAP) flap (9 × 5 cm) and the pectoralis major myocutaneous flap (PMMF) (10×5 cm). Both flaps were transposed to the neck via a subcutaneous tunnel. The TAAP flap reconstructed the posterior cervical esophageal defect, while the PMMF was sutured to its edge to form a tubular structure. This structure was sutured at the upper end to the tongue base and at the lower end to the esophageal defect. This suturing procedure successfully reconstructed the cervical esophagus. An endotracheal tube emerged through the anterior neck skin as a stoma. The donor site wound was primarily closed (Figure 3).

#### Case 2

**Figure 4:**
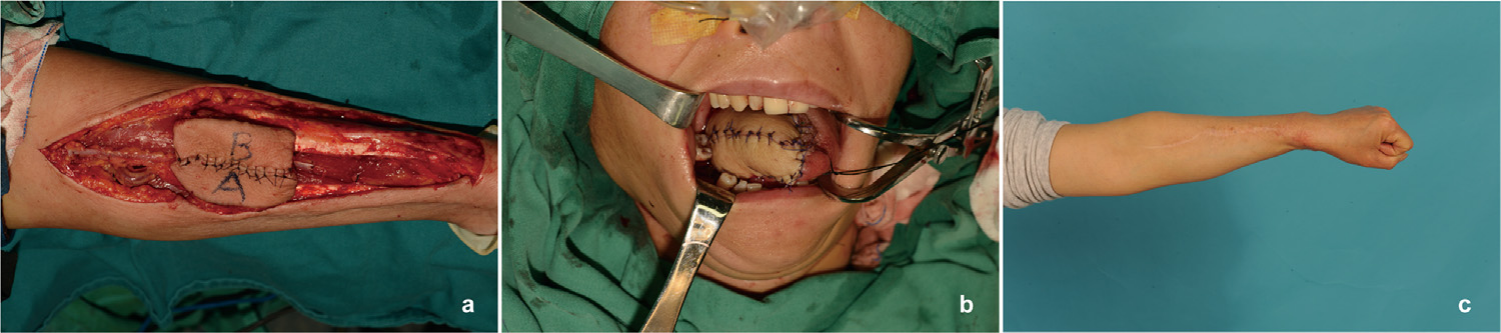
Case 2. Reconstruction of tongue defect using narrow radial forearm free flap with “non-classic” kiss technique. (a) Assembly of wide radial forearm free flap (RFFF) by intermediate de-epithelialization and distal flap rotation splicing. (b) Immediate post-reconstruction appearance. (c) Appearance of the left forearm donor site at 3 years postoperatively.

A 49-year-old woman with T2N0M0 right tongue squamous cell carcinoma underwent right functional neck dissection and hemilingual tumor resection, leaving a 5 × 4.5 cm oral cavity defect. A narrow radial forearm free flap (nRFFF), based on the radial artery and sized 2.5 × 13 cm, was designed. The de-epithelialized central region of the nRFFF was rotated and sutured to form a 6.5 × 5 cm flap, with edges joined to the remaining tongue margin and mouth floor. The vascular pedicle was passed from the mandible’s inner side to the neck. Anastomoses were performed end-to-end: the radial artery to the superior thyroid artery, the radial vein to the internal jugular vein, and the cephalic vein to the external jugular vein using a vascular stapler. The donor site was closed directly without tension (Figure 4).

#### Case 3

**Figure 5:**
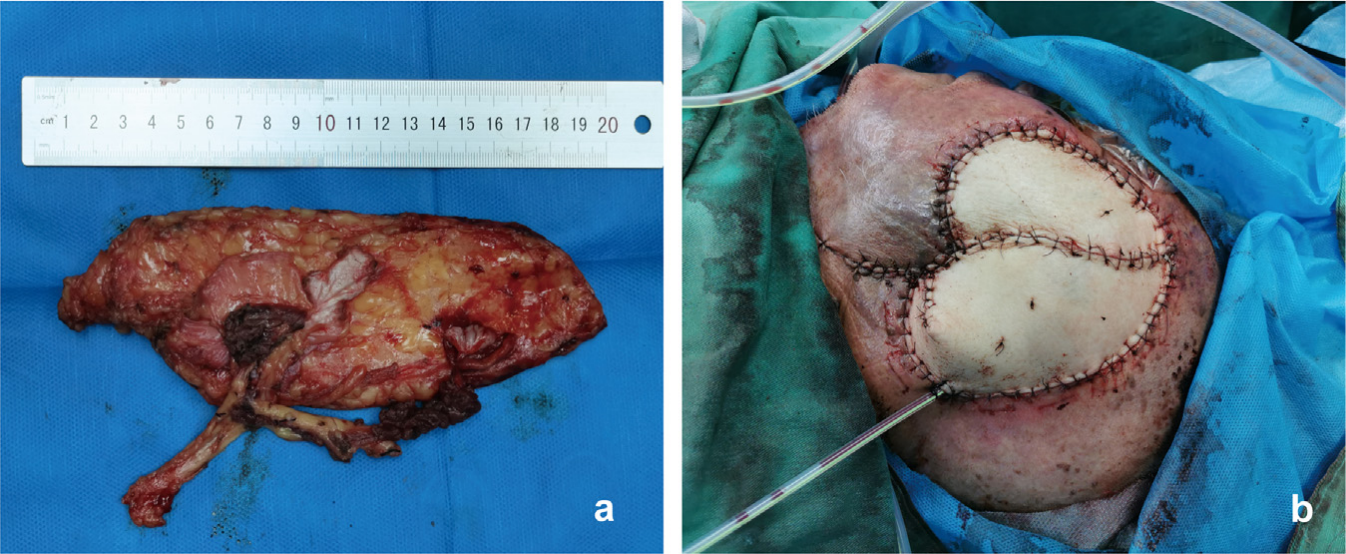
Case 3: Reconstruction of temporal defect using dual-perforator anterolateral thigh (ALT) flap with “classic” kiss technique. (a) Harvesting of a free double-perforator ALT flap. (b) Immediate postoperative appearance of reconstruction using a dual-perforator ALT flap.

A 79-year-old male with a large malignant skin tumor in the left temporal region, staged T4N1M0, was diagnosed with poorly differentiated squamous cell carcinoma. He underwent left functional neck dissection and extended tumor resection, leaving a 12 × 12 cm defect. A double-perforator ALT flap measuring 19 × 6.5 cm was harvested to reconstruct the temporal defect while achieving primary closure of the donor site. Two ALT kiss flaps, 10 × 6.5 cm and 9 × 6 cm, were prepared using the “classic” method. These flaps were rotated and joined by their length-to-width proportions to repair the temporal defect. The flap’s arterial pedicle was anastomosed end-to-end with the facial artery, while the two venous pedicles were separately anastomosed to the facial vein and the proximal internal jugular vein. The leg donor site was closed directly in one stage (Figure 5).

#### Case 4

**Figure 6:**
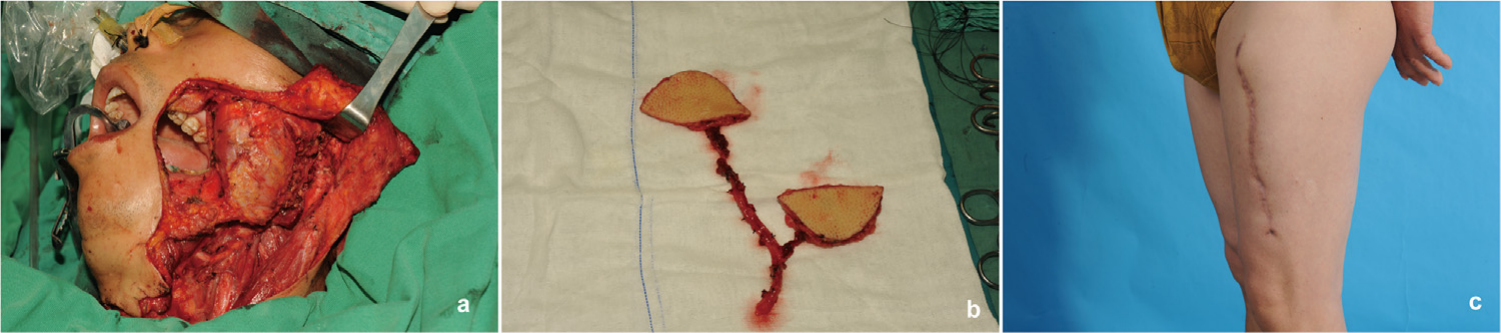
Case 4: Reconstruction of left buccal through-and-through defect using dual-perforator anterolateral thigh (ALT) flap with kiss technique. (a) Through-and-through defect of the left buccal region following tumor resection. (b) Harvesting of double-island ALT flap. (c) Appearance of the thigh donor site at 1 year postoperatively.

A 45-year-old male with T3N1M0 left buccal squamous cell carcinoma underwent radical resection, resulting in a through-and-through defect. To reconstruct this, ALT kiss flaps sized 6.5 × 5 cm and 5 × 4.5 cm with double perforators were prepared using the “classic” method. The larger flap reconstructed the oral mucosal surface, while the smaller flap was used for the facial aspect. The vascular pedicle was superficially routed over the mandible to the neck. The arterial pedicle was anastomosed end-to-end with the superior thyroid artery, and the venous pedicles were connected separately in an end-to-side manner to the left internal jugular vein. The total facial vein was anastomosed end-to-end using a surgical stapler. The thigh donor site was closed in one stage (Figure 6).

## Discussion

For head and neck cancer patients, the significance of postoperative functional and aesthetic reconstruction is increasingly recognized. A primary challenge for reconstructive surgeons is optimizing resource utilization while maintaining quality. Core principles of flap economics involve minimizing donor site morbidity, judicious flap selection, reducing waste through optimized design, and customizing reconstruction plans. These strategies aim to maximize outcomes with limited resources, providing optimal therapeutic results. However, applying these principles in practice presents challenges that require in-depth research.

In recent years, free flap transfer has emerged as the preferred method for large postoperative defects in head and neck oncology.^15^, ^16^, ^17^, ^18^ Nonetheless, pedicled flaps maintain essential roles in specific contexts.^3^^,^^15^^,^^19^^,^^20^ Literature indicates that kiss flaps are primarily harvested using a “classic” approach with multi-perforator flaps.^6^^,^^7^^,^^14^^,^^21^^,^^22^ Additionally, the “non-classic” method is viable for obtaining kiss flaps from single-perforator or musculocutaneous flaps. Studies^23^^,^^24^ have demonstrated the existence of an arteriovenous network in both superficial and deep fascia, ensuring reliable skin perfusion, thereby supporting the safety of the de-epithelialization technique. Both “classic” and “non-classic” methods in harvesting kiss flaps reflect flap economic principles, some on independent perforators, some where the paddles are connected by a de-epithelialized strip. This study, based on experiences from two centers, retrospectively analyzed the efficacy and safety of four standard workhorse flaps and their associated kiss flaps for reconstructing postoperative defects in head and neck cancer, providing a reference for clinical practice.

Although there was no statistically significant difference in complication rates between the classical and non-classical groups, our experience suggests that the classical Kiss flap technique is more versatile in reconstructive applications. Compared to conventional non-Kiss flap techniques, it demonstrated a higher complication rate, which may be attributed to the typically more complex cases managed with the Kiss technique.^17^^,^^18^^,^^22^ The “classic” approach requires specific perforator numbers and blood supply distribution, limiting flap type choices. However, it allows for larger flap areas and flexible anastomosis options, making it ideal for reconstructing diverse head and neck regions. The “non-classic” approach offers more flap type options but limits the flap area and mobility, making it suitable for localized defect reconstruction. Notably, thinning flaps to the fat layer increased necrosis risk compared to simple de-epithelialization, possibly due to excessive thickness, compromised blood supply from compression, or perforator damage during dissection. Literature suggests fascial blood supply is mainly on the fascial surface.^23^^,^^25^ Hence, we advise against dissecting into the fat layer during de-epithelialization and stress preserving blood supply. The elevated complication rates observed in this study may be attributed to three primary factors: ​limited sample size, ​selection bias, and ​technical challenges associated with de-epithelialization techniques.

We categorized postoperative head and neck cancer defects into three types by location: A - skin defects, B - oral and maxillofacial defects, and C - hypopharyngeal and below defects. Type A small skin defects (<3 cm) are typically repaired by direct closure. Adjacent or pedicled flaps suit defects 50–100 cm², while those over 100 cm² benefit from free flap transplantation.^14^^,^^26^^,^^27^ Our study solely used ALT flaps for Type A, likely due to larger defects in late-stage presentations. Multi-perforator ALT with the “classic” method successfully covered large scalp defects, allowing primary closure of the donor site. This method aligned with findings from Zuo et al. ,^22^ showing effective repair and survival.

Type B defects, being more complex, mostly utilized ALT, followed by RFFF. We chose RFFF for small defects with high BMI and ALT or PMMF for larger ones, with Fibula essential for simultaneous mandibular reconstruction. In challenging cases, multiple flap combinations were employed for reconstruction.^8^^,^^12^^,^^28^ Our key insights: ① Larger intraoral flaps prevent trismus in buccal defects, while smaller extraoral flaps offer needed tension and reduced ptosis. ② Multi-perforator flaps enhance survival and aesthetics. ③ Muscle flaps effectively reduce infection and support structure, confirming prior studies.^6^

For Type C defects, restoring digestive tract continuity is crucial. Smaller defects may use RFFF or ALT, while circumferential ones often require tubularized ALT, free jejunal flaps, or gastric pull-up. In vascular-depleted necks or combined hypopharyngeal and skin defects, PMMF with TAAP is recommended.^1^^,^^8^^,^^29^ We used ALT and PMMF in our study, advocating for multi-perforator ALT or PMMF with sub-perforators via the “classic” method and TAAP to safeguard anastomotic vessels and prevent pharyngocutaneous fistula.

Kiss flaps can be harvested by either “classic” or “non-classic” methods, achieving aesthetic and functional reconstruction regardless of the flap used. ALT offers versatility with fasciocutaneous, myocutaneous, perforator, and chimeric types, making it suitable for almost all head and neck regions, such as the scalp, oral cavity, oropharynx, hypopharynx, and skull base. It is currently the most widely used workhorse flap.^8^^,^^30^ Large-scale studies report an ALT flap survival rate of about 97 %.^5^^,^^31^

RFFF is preferred for smaller intraoral and extraoral defects, like the tongue, buccal, and pharyngeal sidewall.^10^^,^^32^ Before 2007, RFFF was commonly used in head and neck reconstructions. However, due to limited volume and the need for skin grafting, which increases complication rates, its usage has declined.^33^^,^^34^ We applied flap economics to prepare nRFFF and a kiss flap, achieving primary closure of the donor site. Literature reviews show 100 % survival in 24 nRFFF cases by Shaikh et al.^35^ and 9 bilobed RFFF cases by Zhang et al.^19^ This suggests nRFFF is still reliable for small defects, especially in patients with higher BMI.

For mandibular reconstruction, the free fibula flap is most common.^11^^,^^36^ Wu et al.^11^ noted higher complication rates due to harvesting complexity, though this remains controversial.^36^ In complex cases, combining flaps like ALT and Fibula can simultaneously reconstruct bone and soft tissue.^8^^,^^11^

PMMF, a common salvage flap, offers reliable blood supply, flexible harvesting, and substantial tissue volume. It can be combined with TAAP for complex defects and is often the last strategy for head and neck oncologic reconstructions.^12^^,^^15^^,^^37^^,^^38^ For circumferential hypopharyngeal defects, PMMF with TAAP is preferred. In our study, two patients were successfully salvaged with PMMF after total flap loss, confirming its reliability.

To achieve satisfactory aesthetic and functional reconstruction, we recommend the following principles: I. Clearly define the extent of the postoperative defect, tissue types needing repair, and reconstruction goals. Choose the most suitable flap based on the patient’s history, preferences, condition, and financial capacity, adhering to flap economics for a tailored plan. II. Conduct thorough physical exams and preoperative assessments using tools like color Doppler, handheld Doppler, CTA, and DSA to evaluate vascular status and perforator locations. III. Ensure meticulous intraoperative procedures, as multi-perforator flap surgeries are complex and prone to perforator injury, requiring careful handling. Select vessels with appropriate diameter and matching conditions for vascular anastomosis. IV. Enhance perioperative management, focusing on postoperative flap monitoring, especially for lobed kiss flaps. Promptly analyze and address any issues. V. Pre-plan rescue strategies for potential complications.

The four flap types in this study are excellent for reconstructing defects post-head and neck tumor resection. Applying flap economics principles, harvesting kiss flaps allowed primary closure of the donor site, achieving better aesthetic and functional outcomes than traditional methods. Optimal outcomes with minimal cost reflect both flap economics and a patient-centered medical model. Patients were sourced from authoritative cancer centers in two Chinese provinces, ensuring data authenticity and representativeness. Although the same techniques were used, different surgical teams may have influenced results. As a retrospective study, bias is challenging to avoid, and the small sample size is a limitation. Future research should explore flap economics in a broader range of flap types through large-sample, multi-center studies.

## Conclusion

Both “classic” and “non-classic” methods for harvesting kiss flaps represent viable alternative options in complicated cases.​ Among the four flap types, the ALT flap stands out for its stability and versatility. Applying flap economic principles enhances resource optimization, while individualized selection of suitable flaps and harvesting techniques is essential for achieving superior aesthetic and functional reconstruction outcomes.

## Funding

Our study was supported by grants from the National Natural Science Foundation of China (81960543, 82360568), and Yunnan Province Basic Research Program (202301AY070001-247).

## Author contributions

WP and HZ conceived and designed the study together with ZL and CS. WP and HZ conducted the investigation and managed the project resources. HZ designed the methodology and performed the formal analysis, while LJ assisted in data analysis and management. WP and HZ drafted the manuscript. All authors contributed to manuscript revision and visualization. CS and WP provided funding support. CS and ZL jointly supervised the project and were responsible for project administration. All authors have reviewed the finalized manuscript and approved the manuscript before submission.

## Ethical approval

Written informed consent was obtained from all individuals depicted in this manuscript for the use and publication of their clinical photographs in accordance with institutional ethical guidelines. This retrospective study was conducted in accordance with the principles of the Declaration of Helsinki and was approved by the Ethics Committee of Yunnan Cancer Hospital (KYLX2024-033).

## Declaration of competing interest

The authors declare no conflicts of interest.

## References

[1] Hanasono M.M., Matros E., Disa J.J. (2014). Important aspects of head and neck reconstruction. Plast Reconstr Surg.

[2] Wong C.H., Wei F.C. (2010). Microsurgical free flap in head and neck reconstruction. Head Neck.

[3] Colletti G., Tewfik K., Bardazzi A. (2015). Regional flaps in head and neck reconstruction: a reappraisal. J Oral Maxillofac Surg.

[4] Zhang Y.X., Hayakawa T.J., Levin L.S., Hallock G.G., Lazzeri D. (2016). The economy in autologous tissue transfer: part 1. The kiss flap technique. Plast Reconstr Surg.

[5] Xu Z., Zhao X.P., Yan T.L. (2015). A 10-year retrospective study of free anterolateral thigh flap application in 872 head and neck tumour cases. Int J Oral Maxillofac Surg.

[6] Yang R., Wu X., Kumar P.A. (2020). Application of chimerical ALT perforator flap with vastus lateralis muscle mass for the reconstruction of oral and submandibular defects after radical resection of tongue carcinoma: a retrospective cohort study. BMC Oral Health.

[7] Wang F., Pradhan P., Li N., Jiang C., Liu W., Zeng L. (2018). Tripaddled anterolateral thigh flap for the reconstruction of extensively full-thickness cheek defects by stacking two skin paddles as kiss pattern. J Craniofac Surg.

[8] Bianchi B., Ferri A., Ferrari S. (2012). The free anterolateral thigh musculocutaneous flap for head and neck reconstruction: one surgeon’s experience in 92 cases. *Microsurg*ery.

[9] Qing L., Wu P., Yu F., Zhou Z., Tang J. (2018). Use of dual-skin paddle anterolateral thigh perforator flaps in the reconstruction of complex defect of the foot and ankle. J Plast Reconstr Aesthet Surg.

[10] Shuck J., Chang E.I., Mericli A.F. (2020). Free lateral forearm flap in head and neck reconstruction: an attractive alternative to the radial forearm flap. Plast Reconstr Surg.

[11] Wu C.C., Lin P.Y., Chew K.Y., Kuo Y.R. (2014). Free tissue transfers in head and neck reconstruction: complications, outcomes and strategies for management of flap failure: analysis of 2019 flaps in single institute. *Microsurg*ery.

[12] Tripathi M., Parshad S., Karwasra R., Singh V. (2015). Pectoralis major myocutaneous flap in head and neck reconstruction: an experience in 100 consecutive cases. Natl J Maxillofac Surg.

[13] Lin Y.S., Liu W.C., Lin Y.S., Chen L.W., Yang K.C. (2017). Peroneal flap for tongue reconstruction. J Reconstr Microsurg.

[14] Xiong L., Guo N., Gazyakan E., Kneser U., Hirche C. (2018). The anterolateral thigh flap with kiss technique for microsurgical reconstruction of oncological scalp defects. J Plast Reconstr Aesthet Surg.

[15] Correia C., Wang W., Vincent A.G., Chan D., Ducic Y. (2020). Regional salvage flap options in head and neck reconstruction. Semin Plast Surg.

[16] Wang W., Ong A., Vincent A.G., Shokri T., Scott B., Ducic Y. (2020). Flap failure and salvage in head and neck reconstruction. Semin Plast Surg.

[17] Sweeny L., Curry J., Crawley M. (2020). Factors impacting successful salvage of the failing free flap. Head Neck.

[18] Ma C., Gao W., Abdelrehem A. (2022). Anteromedial thigh septocutaneous perforator flap as a first choice for head and neck reconstruction: a clinical algorithm based on perforator-pedicle relationship. Oral Oncol.

[19] Zhang Y.X., Xi W., Lazzeri D. (2015). Bipaddle radial forearm flap for head and neck reconstruction. J Craniofac Surg.

[20] Parr J.M., Chouhan P., Wagels M. (2019). Anterolateral thigh versus pectoralis major flaps in reconstruction of the lateral temporal bone defect. ANZ J Surg.

[21] Song D.J., Li Z., Zhang Y.X. (2023). Combination mode and optimization strategy of harvest procedure of anterolateral thigh chimeric perforator myocutaneous flap. Chin J Repar Reconstr Surg.

[22] Zuo L., Yu J.J., Zhou X. (2018). Application of free anterolateral thigh kiss flap in repair of large scalp defect after malignant tumor resection. Chin J Repar Reconstr Surg.

[23] Janik S., Hirtler L., Traxler H., Weninger W.J., Seemann R., Erovic B.M. (2020). The vascularized fascia lata free flap: an anatomical study and clinical considerations. Eur Arch Otorhinolaryngol.

[24] Stecco C., Tiengo C., Stecco A. (2013). Fascia redefined: anatomical features and technical relevance in fascial flap surgery. Surg Radiol Anat.

[25] Mosahebi A., Disa J.J., Pusic A.L., Cordeiro P.G., Mehrara B.J. (2008). The use of the extended anterolateral thigh flap for reconstruction of massive oncologic defects. Plast Reconstr Surg.

[26] Mahmoud W.H. (2021). Single stage reconstruction of large calvarial exposure after tumor resection: a 3-year experience. World J Plast Surg.

[27] Kruse-Lösler B., Presser D., Meyer U., Schul C., Luger T., Joos U. (2006). Reconstruction of large defects on the scalp and forehead as an interdisciplinary challenge: experience in the management of 39 cases. Eur J Surg Oncol.

[28] Liu W.C., Yang K.C. (2015). One-stage through-and-through cheek, lips, and oral commissure reconstruction using a double-paddle peroneal chimeric flap. Head Neck.

[29] Reiter M., Baumeister P. (2019). Reconstruction of laryngopharyngectomy defects: comparison between the supraclavicular artery island flap, the radial forearm flap, and the anterolateral thigh flap. Microsurgery.

[30] Kidd T., Platt N., Kidd D., Grobbelaar A.O. (2022). Short- and long-term complications of free anterolateral thigh flap reconstructions: a single-centre experience of 92 consecutive cases. Surgery Res Pract.

[31] Ren Z.H., Wu H.J., Wang K., Zhang S., Tan H.Y., Gong Z.J. (2014). Anterolateral thigh myocutaneous flaps as the preferred flaps for reconstruction of oral and maxillofacial defects. J Craniomaxillofac Surg.

[32] Liu W.W., Li H., Guo Z.M. (2011). Reconstruction of soft-tissue defects of the head and neck: radial forearm flap or anterolateral thigh flap?. Eur Arch Otorhinolaryngol.

[33] Zhang C., Sun J., Zhu H. (2015). Microsurgical free flap reconstructions of the head and neck region: shanghai experience of 34 years and 4640 flaps. Int J Oral Maxillofac Surg.

[34] Orlik J.R., Horwich P., Bartlett C., Trites J., Hart R., Taylor S.M. (2014). Long-term functional donor site morbidity of the free radial forearm flap in head and neck cancer survivors. J Otolaryngol Head Neck Surg.

[35] Shaikh S.A., Bawa A., Shahzad N., Yousufzai Z., Ghani M.S. (2018). Reducing the donor site morbidity in radial forearm free flaps by utilizing a narrow radial forearm free flap. Arch Plast Surg.

[36] Lonie S., Herle P., Paddle A., Pradhan N., Birch T., Shayan R. (2016). Mandibular reconstruction: meta-analysis of iliac- versus fibula-free flaps. ANZ J Surg.

[37] Gabrysz-Forget F., Tabet P., Rahal A., Bissada E., Christopoulos A., Ayad T. (2019). Free versus pedicled flaps for reconstruction of head and neck cancer defects: a systematic review. J Otolaryngol Head Neck Surg.

[38] Bathula S.S., Stern N.A., Ross A., Patrick T., Talatala E.R. (2021). Role of pectoralis major myocutaneous flap in laryngectomy surgery: single surgeon experience. Cureus.

